# Pixelated Checkerboard Metasurface for Ultra-Wideband Radar Cross Section Reduction

**DOI:** 10.1038/s41598-017-11714-y

**Published:** 2017-09-12

**Authors:** Mohammad-Javad Haji-Ahmadi, Vahid Nayyeri, Mohammad Soleimani, Omar M. Ramahi

**Affiliations:** 1 Antenna and Microwave Research Laboratory, Iran University of Science and Technology, Tehran, 1684613114 Iran; 2 Department of Electrical and Computer Engineering, University of Waterloo, Waterloo, ON N2L3G1 Canada

## Abstract

In this paper we designed and fabricated a metasurface working as a radar cross section (RCS) reducer over an ultra wide band of frequency from 3.8 to 10.7 GHz. The designed metasurface is a chessboard-like surface made of alternating tiles, with each tile composed of identical unit cells. We develop a novel, simple, highly robust and fully automated approach for designing the unit cells. First, a topology optimization algorithm is used to engineer the shape of the two unit cells. The area of each unit cell is pixelated. A particle swarm optimization algorithm is applied wherein each pixel corresponds to a bit having a binary value of 1 or 0 indicating metallization or no metallization. With the objective of reducing the RCS over a specified frequency range, the optimization algorithm is then linked to a full wave three-dimensional electromagnetic simulator. To validate the design procedure, a surface was designed, fabricated and experimentally tested showing significantly enhanced performance than previous works. Additionally, angular analysis is also presented showing good stability and wide-angle behavior of the designed RCS reducer. The automated design procedure has a wide range of applications and can be easily extended to design surfaces for antennas, energy harvesters, noise mitigation in electronic circuit boards amongst others.

## Introduction

Metasurfaces are electrically-thin structures often manufactured by patterning a periodic ensemble of conducting shapes on a dielectric host medium. These structures have attracted high attention since they create specific and unusual electromagnetic (EM) properties enabling new technologies and addressing key challenges in various applications^1–8^. As an important feature of metasurfaces, they reflect an incident EM wave with a phase difference which varies, depending on the specific design, between +180° and −180° as function of frequency. This unique property paved the way for engineering surfaces with a desired reflection phase which has recently been shown to enable controlling the direction of the scattered fields^9–16^.

In stealth technology, a key objective is to reduce the radar cross section (RCS) of metallic objects such as missile and aircrafts. Basically, there are two main ways to achieve this objective. In the first approach, the object is covered with a radar absorbing material (RAM) converting the incident EM energy into heat^17–19^. Although, applying RAM has certain advantages, it has drawbacks including high overall material thickness, heavy weight, and high material and maintenance cost. In addition, in most cases RAM is inherently narrowband^20–22^. The second approach entails redirecting the scattered energy away from the radar receiver. This approach is traditionally implemented by controlling the geometrical features of the object at considerable cost as it also involves reconsideration of non-electromagnetic aspects of the design such as aerodynamics and mechanical integrity amongst others.

**Figure 1:**
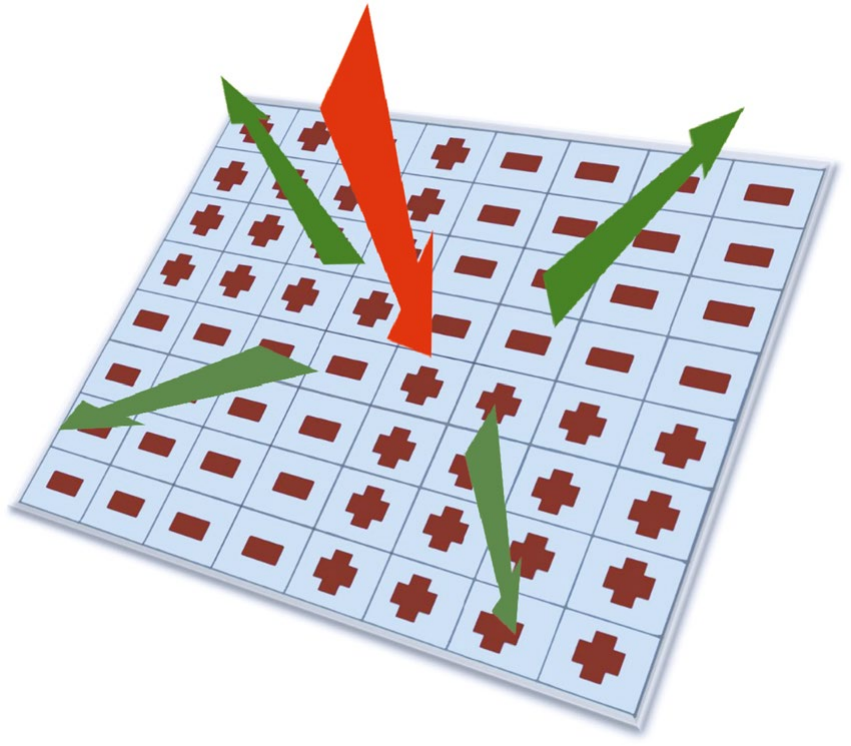
Redirecting EM wave by a chessboard-like configuration formed by combining two AMC cells.

Recently, applying metasurfaces has been proposed as an effective technique for redirecting the scattered field by using a chessboard-like surface composed of artificial magnetic conductor (AMC) and perfect electric conductor (PEC) cells as a covering layer of a metallic object^23^. Due to in-phase and out-of-phase reflections from adjacent PEC and AMC cells, the scattered fields are altered and the energy is redirected without changing the shape of the underlying object. The advantage of this technique is that the covering structure is thin, low profile and low cost; however, the RCS reduction occurs over a very narrow bandwidth of approximately 5%^23^. To overcome this problem, a chessboard-like configuration formed by combining two AMC cells (as shown in Fig. 1) has been proposed, designed and fabricated^24–26^. In the chessboard-like surface concept, two AMC cells were designed to provide almost out-of-phase reflections (i.e., phase difference of 180 ± 30°) over a wider frequency bandwidth. In order to achieve a broader bandwidth, various works focused on the design of the cells in the chessboard-like structure^27–29^. In these studies, several unit cells with simple traditional shapes such as a square patch, a circular patch, and the Jerusalem cross were applied and their geometrical dimensions were optimized to maximize the bandwidth. In other works, instead of designing two unit cells and then forming a regular checkerboard surface as shown in Fig. 1, several unit cells providing different reflection phases are designed and then by an irregular formation of the cells a RCS reducer surface is achieved^30^. However, the drawback of this strategy is that if the shape or dimensions of the underlying object are changed, the covering surface should be designed again.

The chessboard-like surface has proven to be a powerful concept that is based on generating secondary sources (from the scatterers) that are expected to provide phase cancellation at electrically far distance from the surface. Ideally, one would expect that a PEC-PMC tiles combination to give the optimal solution. However, as previous publications have showed, this is not the case. In other words, not only the overall far-field phase is important but also the physical interaction of the near-fields is critical in mitigating the far field radiation. Which unit cell design, within each tile, provides maximum bandwidth is the main drive behind this paper. Such problem, to the best of the authors knowledge, cannot be posed analytically. Therefore, in this work, we develop a complete-cycle procedure to design the unit cell that gives optimal performance over a specified frequency band. Here, optimality is defined with respect to the specific optimization methodology used in this work.

## Unit Cell Design Methodology

Figure 1 shows a checkerboard-like surface composed of alternating tiles formed by two different types of unit cells such that each tile consists of 4 × 4 identical cells. Suppose that the surface is illuminated by a plane wave incident in a direction normal to the surface. If the reflections from the alternating tiles are out of phase, there will be cancellation in the direction normal to the surface, thus implying reduced reflected field. It is theoretically proven that a phase difference of 180 ± 37° between the reflections from two region provides at least 10 dB monostatic RCS reduction^28^. For this end, two unit cells should be designed in a way that the reflections from the alternating tiles are almost out of phase. Since the structure is hosted by a low-loss dielectric substrate and backed by a metallic layer, the reflection amplitude is near unity. Since each tile consists of N × N cells (where N ≥ 4 in the example shown in Fig. 1), the reflection phase from each tile can be approximated by that from an infinite periodic structure of composed of identical cells. This approximation allow for efficient simulation by using a periodic boundary condition (PBC) applied only on one unite cell.

**Figure 2:**
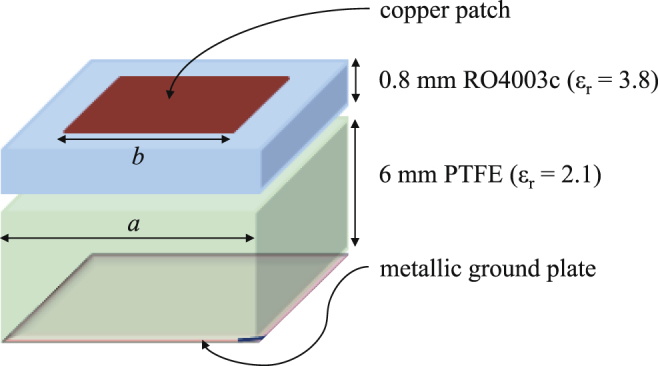
A simple unit cell formed by printing a square patch on a two-layer dielectric substrate. The substrate is backed by a metallic sheet.

Figure 2 shows a simple unit cell formed by printing a conducting square patch on a dielectric substrate. The size of the unit cell, the shape and the size of the conducting patch, and the dielectric constant and the thickness of the substrate all directly affect the reflection phase. It was shown that for lower operating frequency, a thicker substrate is needed^1, 28^. In this work, the desired operating frequency band is 3 GHz to 11 GHz, therefore a dielectric substrate having a thickness of 6 mm is required. However, PCB substrates thicker than 2.5 mm are not widely available. To overcome this problem, we used two-layers substrates of Rogers RO4003c and Teflon (PTFE) with dielectric constant of 3.38 and 2.2, respectively. The conducting patches are printed on a Rogers RO4003c board with thickness of 0.8 mm, then the board is glued on a PTFE sheet with thickness of 6 mm as shown in Fig. 2. The bottom surface of Teflon is covered with aluminum adhesive serving as the metallic backing.

### Preliminary Design

**Figure 3:**
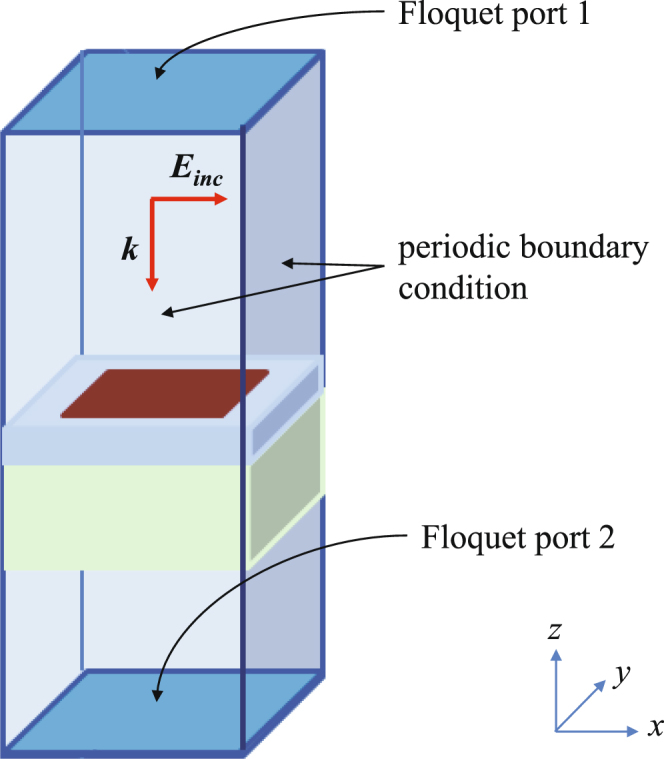
Setup used for full-wave simulation of unit cells in the CST microwave studio.

**Figure 4:**
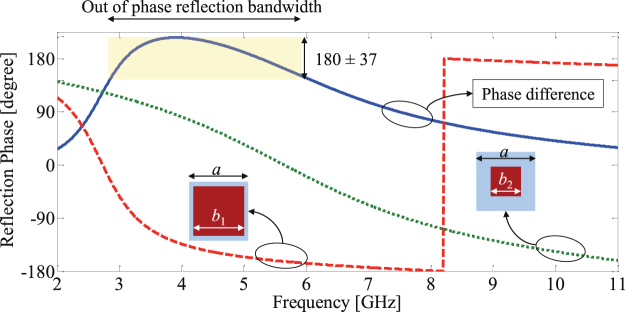
Reflection phase from two unit cells formed by printing square patches on a two-layer dielectric substrate. *a* = 14 mm, *b*_1_ = 12.8 mm and *b*_2_ = 6.4 mm.

Without loss of generality and for simplification of the design process, we consider the two cell sizes (where each cell is the constitutive element for each tile) to be identical. Next, the size of the square patch (see Fig. 2) for each cell is tuned using a simple trial-and-error procedure to maximize the bandwidth over which the reflections from the infinite periodic structures corresponding to each unit cells have a 180 ± 37° phase difference. It should be noted that our final objective is to maximize the operating bandwidth over the frequency range of 3 GHz to 11 GHz. EM simulations are carried out using the frequency domain solver of the CST microwave studio^36^ by applying a periodic boundary condition and exciting the cell with Floquet ports as illustrated in Fig. 3. The values of the variables *a*, *b*_1_ and *b*_2_ were obtained as *a* = 14 mm, *b*_1_ = 12.8 mm and *b*_2_ = 6.4 mm. As indicated in Fig. 4, the reflections from two periodic structures composed of these unit cells provide the desired phase difference (180 ± 37°) in the frequency range of 2.8 to 6.1 GHz, corresponding to a 74% fractional bandwidth. We emphasize that our goals is to achieve a bandwidth of 3–11 GHz. This preliminary design is intended purely as an initial design for the PSO routine that will be discussed next.

### Topology Optimization

**Figure 5:**
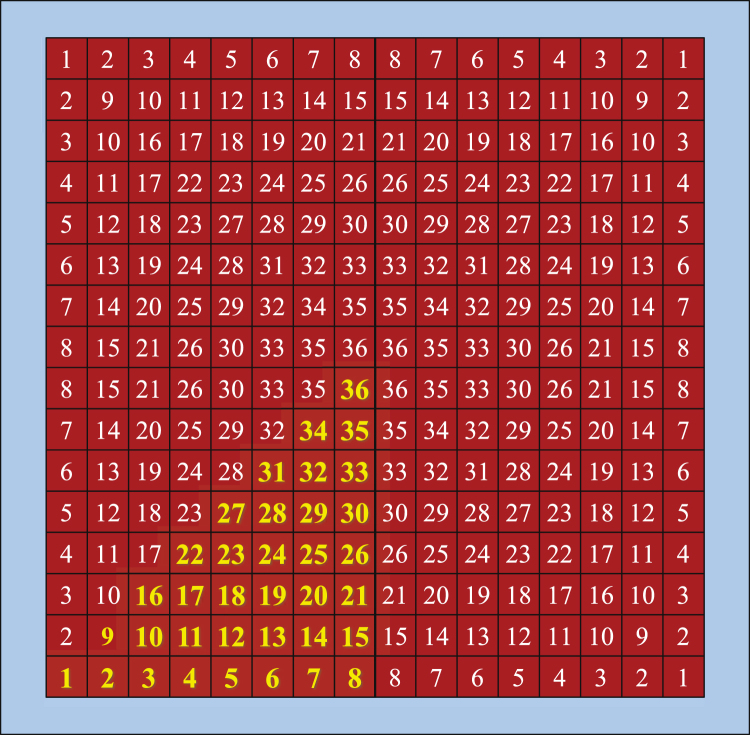
A schematic showing the pixelation of a square patch. The patch is divided into 256 pixels but only 36 pixels are independent (the pixels highlighted in yellow) to achieve geometric symmetry with respect to the lines that bisect the structure vertically, horizontally and diagonally.

To obtain wider bandwidth, we employ an optimization routine to obtain the best possible shape of the copper patch for each unit cell (instead of a square shape). To this end, we first assume the overall sizes of the conductive patches are fixed to 12.8 × 12.8 mm^2^ and 6.4 × 6.4 mm^2^, which were obtained in the preliminary design for the two unit cells. Then the area of each square patch is divided into 16 × 16 = 256 pixels. A binary optimization algorithm is applied wherein each pixel is represented as a bit having a binary value of 1 or 0 indicating the presence or absence of copper on the top of dielectric substrate. To reduce the sensitivity of the structure to the polarization of the incident wave, full geometric symmetry (with respect to the lines that bisect the structure vertically, horizontally and diagonally) is enforced in the design of the unit cells. Therefore, as shown in Fig. 5, only the pixels located in one-eighth of the meshed areas (i.e., a total of 36 pixels for each cell) are considered in the optimization procedure. All other cells will be mirror images of these cells.

The primary optimization procedure was based on binary particle swarm optimization (BPSO) code^31–33^. PSO is a global optimization algorithm finding the best solution of a problem by minimizing a cost function^34^. The particles in the PSO (and BPSO) are the population of candidate solutions. The particles move around in the search space according to simple mathematical formulae that describe the particles’ position and velocity. The movement of each particle is influenced by the local best known solution experienced by the particle, but is also guided toward the global best known solution founded from other particles. It is expected that the particles converge to the global best solution. More details about the optimization algorithm are provided in the Method section. The BPSO was applied to a string of 36 bits for each unit cell (a total of 72 bits were used in the code) to maximize the bandwidth of the out-of-phase reflection. To accelerate the convergence of the optimization algorithm, the initial values of the bits were set to 1 meaning that the initial shapes of the copper patches were those obtained from the preliminary design.

The cost function is defined as:

1 $$CF=\frac{100}{FBW}$$

where *FBW* is the fractional bandwidth in which the reflection coefficients of two unit cells have a phase difference in the range of 180 ± 37°

2 $$FBW=\frac{100({f}_{max}-{f}_{min})}{0.5({f}_{max}+{f}_{min})}$$

**Figure 6:**
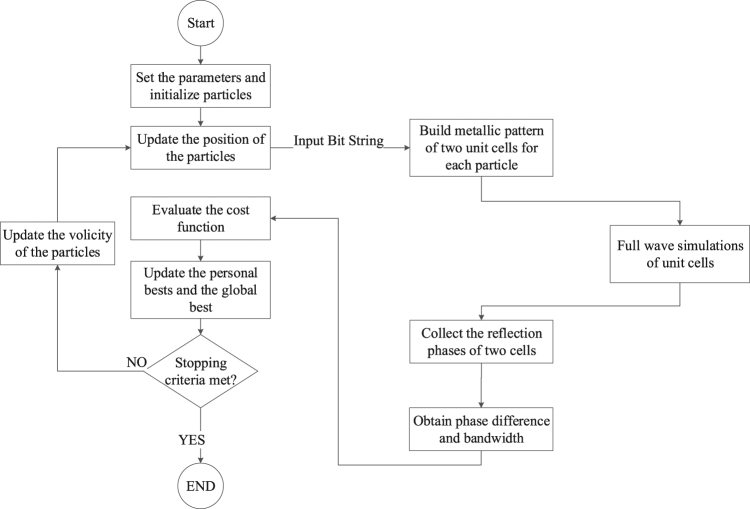
Flowchart of the unit cell topology optimization process.

To determine the *FBW*, the MATLAB optimization code is linked to the CST full-wave EM simulator. The design procedure flowchart is detailed in Fig. 6. The output of the optimization code is a string of bits. Subsequently, the two unit cells with metallic patterns corresponding to the string are built. As shown in Fig. 3, by applying a periodic boundary condition and exciting the unit cell with Floquet ports, the frequency domain solver of CST obtains the reflection coefficient of each unit cell in the frequency range of 3 GHz to 11 GHz. A data process module which is written in MATLAB collects the reflection coefficients, calculates the phase difference between them as a function of frequency, and finally determines the fractional bandwidth.

**Figure 7:**
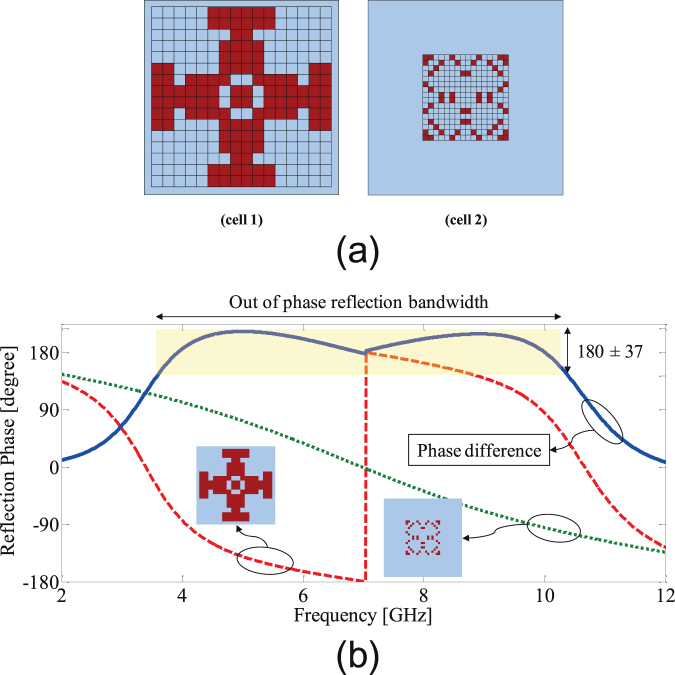
(**a**) Optimized layout of the unit cells and (**b**) reflection phase from two unit cells.

By setting the swarm size of BPSO to 100, after approximately 200 iterations, the algorithm converged. The optimal values of the bits are

3 $$\beta =\{\begin{array}{c}000001110000111000001000111011011101\\ 110001000010010100000000010000001000\end{array}\}$$

where the first and second lines of the string indicate the pixels of the first and second unit cells, respectively (the pixels are numbered in Fig. 5). The optimized cells are shown in Fig. 7(a). It is important to keep in mind that while the unit cell size is uniform, the pixelation is not identical for cell 1 and cell 2 as shown in Fig. 7(a).

The copper patch of these cells clearly do not have simple shapes such as squares, circles or crosses; however as plotted in Fig. 7(b) these two cells, when incorporated into the chessboard (tiles) structures, provide a phase difference of 180 ± 37° between their reflections in the frequency range of 3.6 GHz to 10.4 GHz, corresponding to a 97% fractional bandwidth. Therefor, by applying the topology optimization described, the out-of-phase reflection bandwidth is significantly increased from 74%, obtained from the preliminary design, to 97%.

## AMC Checkerboard Design and Simulation Results

**Figure 8:**
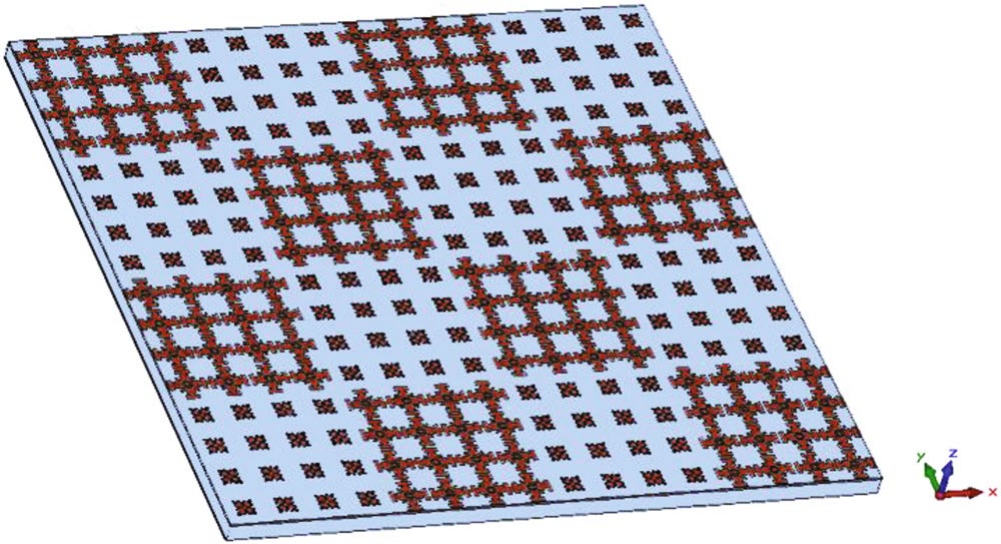
Schematic of the designed RCS reducer surface.

Once two unit cells providing out-of-phase reflections are designed, as shown in Fig. 8, a checkerboard-like metasurface is formed by 4 × 4 alternating AMC tiles where each tile consists of 4 × 4 identical unit cells. Considering that the size of each unit cell is 14 mm × 14 mm, the overall size of the RCS reducer surface is 224 mm × 224 mm.

**Figure 9:**
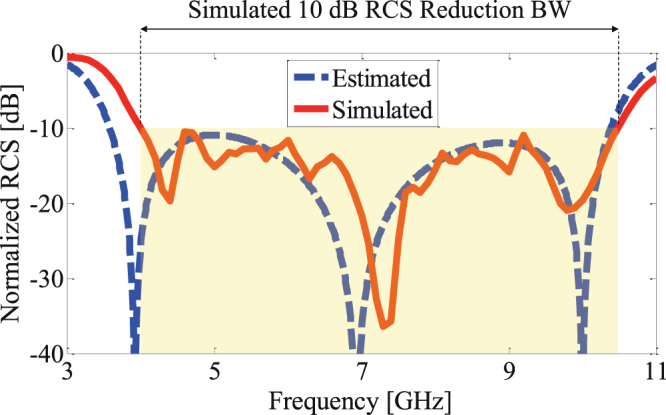
Simulated and estimated monostatic RCS of the RCS reducer surface normalized with respect to that of an equal size metallic plate.

Under the assumption of normal plane wave incidence, due to phase cancellation between the reflected waves from alternating AMC tiles, in the scattering pattern, the main reflected lobes are redirected from the direction of the incoming wave. Two approaches are considered here to analyse the electromagnetic scattering from the surface. In the first approach, the reflection from each AMC tile is approximated as the reflection due to an infinite periodic array of the composer unit cell of the tile. Applying this approach, the RCS reduction of the chessboard surface, compared to a PEC surface (in the direction normal to the surface), can be approximated by^28^

4 $${\rm{RCS}}\,{\rm{Reduction}}=10\,\mathrm{log}[\frac{|{{\rm{\Gamma }}}_{1}\mathrm{|.}\,{{\rm{e}}}^{j\angle {{\rm{\Gamma }}}_{1}}+|{{\rm{\Gamma }}}_{2}\mathrm{|.}\,{{\rm{e}}}^{j\angle {{\rm{\Gamma }}}_{2}}}{2}],$$

where ${{\rm{\Gamma }}}_{1}=|{{\rm{\Gamma }}}_{1}|{{\rm{e}}}^{j\angle {{\rm{\Gamma }}}_{1}},{{\rm{\Gamma }}}_{2}=|{{\rm{\Gamma }}}_{2}|{{\rm{e}}}^{j\angle {{\rm{\Gamma }}}_{2}}$ are the reflection coefficients from the infinite periodic array of unite cell 1 and unit cell 2, respectively. (Notice that, $|{{\rm{\Gamma }}}_{1}|\approx |{{\rm{\Gamma }}}_{2}|\approx 1$ and the reflection phases are given in Fig. 7(c)). The RCS reduction calculated by (4) is shown in Fig. 9 by the blue dashes line. To have a more accurate analysis, the finite RCS reducer surface (exactly as shown in Fig. 8) is simulated using a full-wave simulation (time domain solver of CST microwave. The full-wave simulation result is also shown in Fig. 9 by the solid red line. Clearly, the results are in an strong agreement. Furthermore, Fig. 9 demonstrates a bandwidth from 4 to 10.5 GHz (90%). When compared to a metallic plate with identical size, the monostatic RCS of the RCS reducer surface is 10 dB lower. The maximum RCS reduction of 36.5 dB occurs at 7.3 GHz.

**Figure 10:**
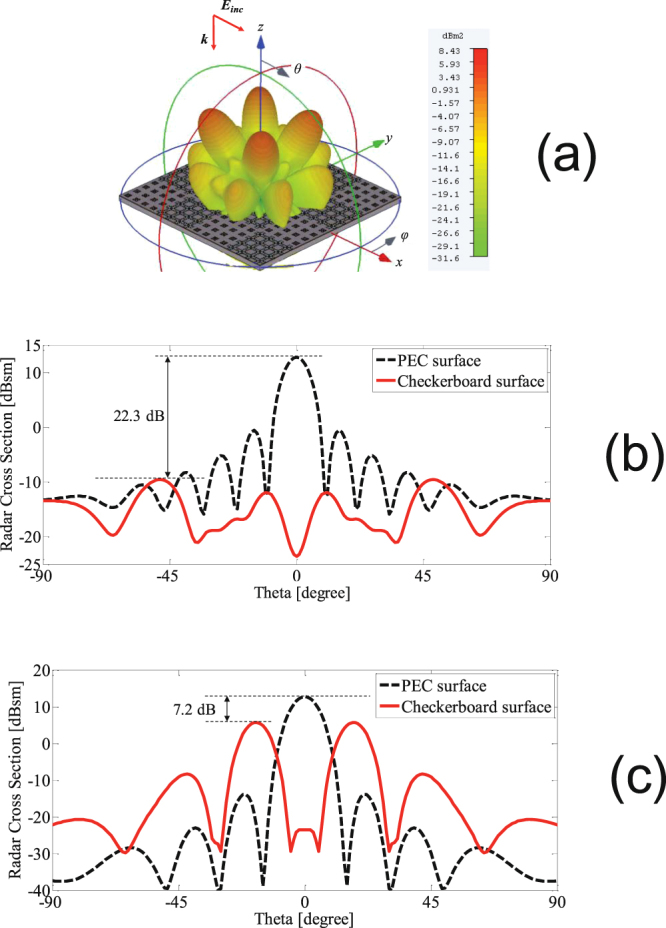
(**a**) 3-D pattern of scattered field from the RCS reducer surface, (**b**) RCS of the in the principal plane (*ϕ* = 0°), and (**c**) RCS in the diagonal plane (*ϕ* = 45°). All the results are for 7.3 GHz.

The 3-D pattern of scattered field from the RCS reducer surface at the frequency where the maximum RCS reduction is achieved (7.3 GHz) is depicted in Fig. 10(a). Clearly, appreciable RCS reduction is achieved in the YZ and XZ planes (*ϕ* = 0°, 90°). The simulation result shows that the main reflected lobes are redirected in the diagonal planes (*ϕ* = 45°, 135°, 225°, 315°) at elevation of *θ* = 20°. At 7.3 GHz, the RCS versus elevation angle (*θ*) in *ϕ* = 0° and *ϕ* = 45° planes is plotted in Fig. 10(b) and (c), respectively, and are compared to those of a same size metallic plate. As shown in Fig. 10(b) and (c), the maximum RCS of the RCS reducer surface in the principal (*ϕ* = 0°) and diagonal (*ϕ* = 45°) planes is 22.3 dB and 7.2 dB lower, respectively, than the maximum RCS of the metallic plate.

**Figure 11:**
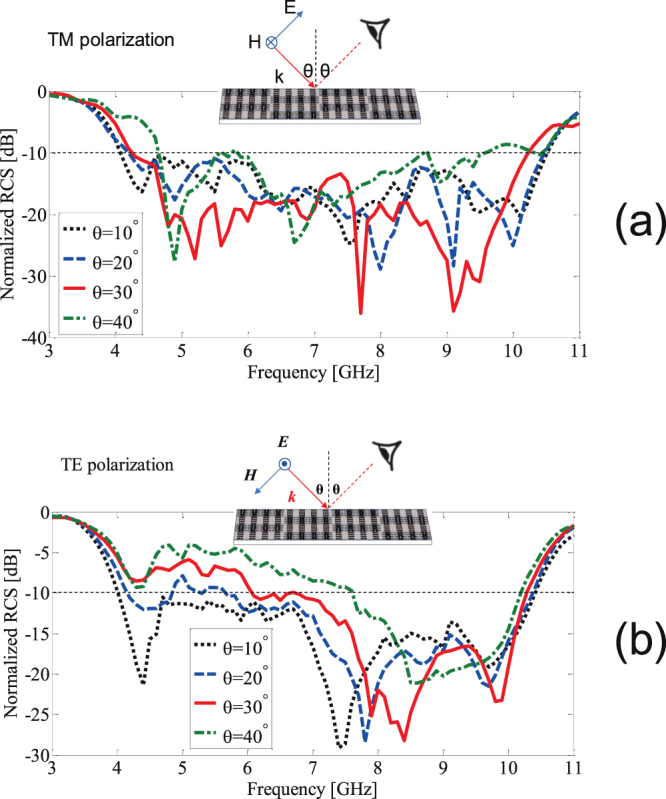
Normalized RCS (with respect to a PEC place) of the optimized RCS reducer surface for oblique incidence. (**a**) TM incident polarization. (**b**) TE incident polarization.

Figure 11(a) and (b) show the performance of the optimized RCS reducer surface under oblique incidence at different incident angles for TM and TE polarization. To gain insight into the performance of the designed surface, the fractional bandwidth of 10-dB RCS reduction is extracted from Fig. 11(a) and (b) and given in Table 1. From Fig. 11 and Table 1, it is obvious that in the case of TM polarized incident wave, the surface works well for a wide range of incident angles; however, in the case of TE polarized incident wave, the operation bandwidth of the board decreased as the incident angle increased. Here we notice that the high sensitivity to the incident angle for the case of TE polarized incident wave is not only a challenge for our design but also for the other designs reported in the literature^27–29^.

**Table 1:** RCS reduction bandwidth for different incident angles.

Incident Angle (*θ*)	10-dB RCS Reduction Fractional Bandwidth	
	TM-Polarization	TE-Polarization
10°	88%	89%
20°	85%	60%
30°	82%	52%
40°	70%	30%

## Experimental Verification

**Figure 12:**
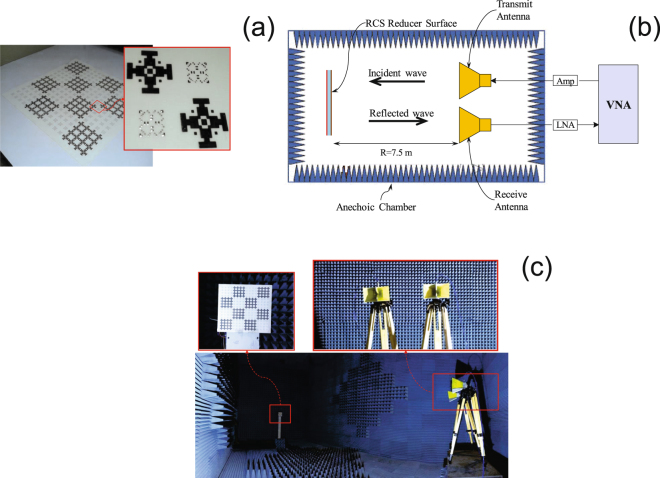
(**a**) Fabricated RCS reducer metasurface. (**b**) Schematic of the measurement setup. (**c**) Experimental measurement setup in an anechoic chamber.

**Figure 13:**
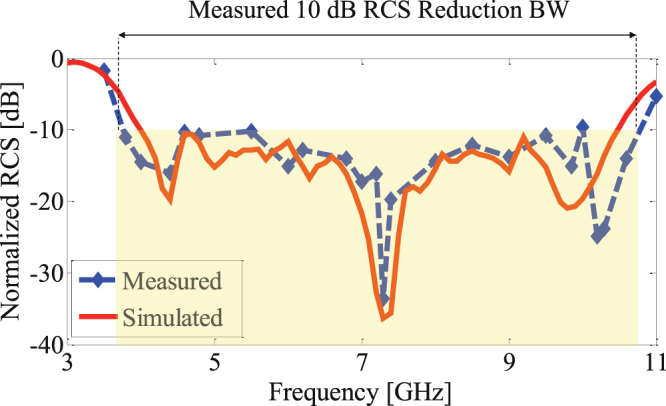
Measured and simulated monostatic RCS of the RCS reducer surface normalized with respect to an equal size metallic plate.

Figure 12(a) shows the fabricated RCS reducer surface composed of 4 × 4 alternating tiles. The monostatic RCS reduction of the fabricated surface under normal incidence of a plane wave was measured in an anechoic chamber. Comparison is made to the response of an equal size PEC surface. The measurement setup used is shown in Fig. 12(b). The fabricated board was supported on top of a polystyrene column in one side of the anechoic chamber. Two identical wideband horn antennas covering the frequency band of 1 to 18 GHz were placed in the opposite side of the chamber as transmitting and receiving antennas. The distance between the transmitting/receiving antennas and the RCS reducer surface was set to 7.5 meters to avoid near field effects. The transmitting and receiving antennas were connected to a vector network analyzer. A measurement reference was established by measuring the reflection from a metal plate with the same size as the RCS reducer surface. Figure 13 shows the measured RCS of the fabricated board with respect to the metal plate which is also compared to the full wave simulation. Strong agreement is observed between measurements and simulation. We note that the measured 10 dB RCS reduction bandwidth (from 3.8 to 10.7 GHz corresponding to a 95% fractional bandwidth) is slightly broader than that obtained by simulation. In addition, it should be noted that there is no difference between the vertical and horizontal polarization of the incident field due to the symmetry of the cells, the pattern and the surface.

## Discussion and Conclusion

A comparison between the performances of the state-of-the-art RCS reducer regular checkerboard surfaces under normally incident plane wave is provided in Table 2. The comparison between the 10 dB RCS reduction bandwidth and the fractional bandwidth clearly shows that our optimized design approach considerably enhanced the operating bandwidth. Furthermore, Table 3 gives a comparison between the 10-dB bistatic RCS reduction bandwidth of the RCS reducer surfaces under an oblique incidence at 30° demonstrating an enhancement of the bandwidth. Hence, by applying a binary optimization algorithm and linking it to a full-wave simulation package, we developed a simple, robust and fully automated unit cell design approach. The simulation and experimental results showed that the RCS reducer surface designed provides excellent broadband properties in a way that its 10 dB RCS reduction bandwidth is appreciably wider than those of earlier works. Finally, even though the surface was not specifically designed for bistatic RCS reduction, full wave simulations showed that the optimized surface not only reduced the monostatic RCS under normal incidence of a plane wave, but also reduced the bistatic RCS under oblique incidence in a bandwidth wider than those reported in earlier works.

**Table 2:** Comparison of 10-dB monostatic RCS reduction bandwidth with similar works.

Reference	Shape of Unit Cells	10-dB monostatic RCS Reduction Bandwidth [GHz]	Fractional Bandwidth [%]
26	Jerusalem Crosses	14.4–21.8	41
27	Patch-Patch and Loop-Cross	9–15	50
25	Patch and Loop	13.25–24.2	58.4
28	Patch and Disk	4.1–7.85	63
**This Work**	**Pixelated Cells**	**3.8**–**10.7**	**95**

**Table 3:** Comparison of 10-dB bistatic RCS reduction bandwidth under oblique incidence with similar works.

Reference	Fractional Bandwidth for 30° Incident Angle	
	TM-Polarization	TE-Polarization
26	20%	Not Reported
28	Not Reported	45%
27	52%	48%
**This Work**	**82%**	**52%**

Notice: ref. 25 did not report result for oblique incidence.

## Methods

### Binary particle swarm optimization (BPSO)

Initially developed in 1995 by Kennedy and Eberhart^34^, particle swarm optimization (PSO) is a search algorithm based on the social behavior of a swarm, such as birds or bees. This algorithm was compared to well known optimization techniques such as the genetic algorithm (GA) in the literature. According to^35^, the convergence of PSO is faster than that of GA which makes it a good candidate for optimization problems which include large number of parameters. PSO works by having a population (called a swarm) of candidate solutions (called particles) which move around the search space. Assuming that the search space is *N*-dimensional (i.e. the number of optimization parameters is *N*), the position and vorticity of m^th^ particle are represented by two N-dimensional vectors ${{\bf{X}}}_{m}=[{x}_{m\mathrm{,1}},{x}_{m\mathrm{,2}}\mathrm{,...,}{x}_{m,N}]$ and ${{\bf{V}}}_{m}=[{v}_{m\mathrm{,1}},{v}_{m\mathrm{,2}}\mathrm{,...,}{v}_{m,N}]$, respectively. Notice that each particle’s position is a candidate solution of the problem. Each particle adjusts its velocity according to its own experience and the best one explored by the swarm (i.e., the experiences of all other particles) in the search space. Hence, at t^th^ iteration of the algorithm, the vorticity of the m^th^ particle is updated by

5 $${{\bf{V}}}_{m}^{t}=w{{\bf{V}}}_{m}^{t-1}+{c}_{1}{\eta }_{1}({{\bf{P}}}_{m}^{t-1}-{{\bf{X}}}_{m}^{t-1})+{c}_{2}{\eta }_{2}({{\bf{G}}}^{t-1}-{{\bf{X}}}_{m}^{t-1}),$$

where ${{\bf{P}}}_{m}=[{p}_{m\mathrm{,1}},{p}_{m\mathrm{,2}}\mathrm{,...,}{p}_{m,N}]$ (the personal best of the m^th^ particle) and ${\bf{G}}=[{g}_{1},{g}_{2}\mathrm{,...,}{g}_{N}]$ (the global best) are the best experiences for the m^th^ particle and the swarm, respectively, *w* is the inertia coefficient, *c*_1_ and *c*_2_ are positive constants and *η*_1_ and *eta*_2_ are random coefficients between 0 and 1 to ensure the random behavior of the optimization algorithm. To further accelerates the convergence, *w* varies from 0.9 at the beginning to 0.4 toward the end of the optimization and the suggested value for *c*_1_ and *c*_2_ is 2^33^.

In a binary search space (where the optimization parameters are assigned a binary value of 0 or 1), each bit *x*_*m*,*n*_ is binary-valued; while, according to 5, *v*_*m*,*n*_s are real-valued. To map the real-valued of the velocities to the range of [0, 1], in the binary version of the PSO algorithm, namely BPSO^31–33^, the sigmoid limiting transformation is used as the following:

6 $$S({v}_{m,n}^{t})=\frac{1}{1+{{\rm{e}}}^{-{v}_{m,n}^{t}}},$$

Finally the position of the m^th^ particle at t + 1^th^ iteration is updated as

7 $${x}_{m,n}^{t+1}=\{\begin{array}{cc}\mathrm{1,} & {r}_{m,n}^{t} < S({v}_{m,n}^{t})\\ \mathrm{0,} & {r}_{m,n}^{t}\ge S({v}_{m,n}^{t})\end{array},$$

where ${r}_{m,n}^{t}$ is a random number with a uniform distribution in (0, 1). The iteration of the algorithm continues until the maximum iterations or minimum error criteria is met. The flowchart of BPSO algorithm is shown in Fig. 6.

## References

[1] Engheta, N. & Ziolkowski, R. W. *Metamaterials: physics and engineering explorations* (John Wiley & Sons, 2006).

[2] Smith DR, Pendry JB, Wiltshire MCK (2004). Metamaterials and negative refractive index. Science.

[3] Alù A (2009). Mantle cloak: Invisibility induced by a surface. Phys. Rev. B..

[4] Padooru YR, Yakovlev AB, Chen P, Alu A (2012). Analytical modeling of conformal mantle cloaks for cylindrical objects using subwavelength printed and slotted arrays. J. Appl. Phys..

[5] Maci S, Minatti G, Casaletti M, Bosiljevac M (2011). Metasurfing: addressing waves on impenetrable metasurfaces. IEEE Trans. Ant. & Prop..

[6] Moser HO, Casse BDF, Wilhelmi O, Saw BT (2005). Terahertz response of a mircofabricated rod-split-ring-resonator electromagnetic metamaterial. Phys. Rev. Lett..

[7] Chen PY, Alù A (2011). Mantle cloaking using thin patterned metasurfaces. Phys. Rev. B..

[8] Guo Y, Yan L, Pan W, Shao L (2016). Scattering engineering in continuously shaped metasurface: An approach for electromagnetic illusion. Sci. Rep..

[9] Yang, F. & Rahmat-Samii, Y. *Electromagnetic band gap structures in antenna engineering* (Cambridge, 2009).

[10] Chen P, Monticone F, Alù A (2011). Suppressing the electromagnetic scattering with a helical mantle cloak. IEEE Ant. Wirel. Prop. Lett..

[11] De Cos ME, Alvarez Y, Las-Heras F (2010). A novel approach for RCS reduction using a combination of artificial magnetic conductors. Prog. Electromagn. Res..

[12] Yang XM, Zhou XY, Cheng Q, Ma HF, Cui TJ (2010). Diffuse reflections by randomly gradient index metamaterials. Opt. Lett..

[13] Wang K, Zhao J, Cheng Q, Dong DS, Cui TJ (2014). Broadband and broad-angle low-scattering metasurface based on hybrid optimization algorithm. Sci. Rep..

[14] Liu Y, Hao Y, Li. K, Gong S (2015). Wideband and polarization independent radar cross section reduction using holographic metasurface. IEEE Ant. Wirel. Prop. Lett..

[15] Song YC, Jun-Ding, Guo CJ, Yu-Hui Ren YH, Zhang JK (2015). Ultra-broadband backscatter radar cross section reduction based on polarization-insensitive metasurface. IEEE Ant. Wirel. Prop. Lett..

[16] Shang Y, Shen Z (2017). Polarization-independent backscattering enhancement of cylinders based on conformal gradient metasurfaces. IEEE Trans. Ant. & Prop..

[17] Kim HK, Lee D, Lim S (2016). Wideband-switchable metamaterial absorber using injected liquid metal. Sci. Rep..

[18] Costa F, Monorchio A, Manara G (2010). Analysis and design of ultra-thin electromagnetic absorbers comprising resistively loaded high impedance surfaces. IEEE Trans. Ant. & Prop..

[19] Kazemzadeh A, Karlsson A (2010). Multilayered wideband absorbers for oblique angle of incidence. IEEE Trans. Ant. & Prop..

[20] Landy NI, Sajuyigbe S, Mock JJ, Smith DR, Padilla WJ (2008). Perfect metamaterial absorber. Phys. Rev. Lett..

[21] Watts CM, Liu X, Padilla WJ (2012). Metamaterial electromagnetic wave absorbers. Adv. Mater..

[22] Luukkonen O, Costa F, Simovski CR, Monorchio A, Tretyakov SA (2009). A thin electromagnetic absorber for wide incidenceangles and both polarizations. IEEE Trans. Ant. & Prop..

[23] Paquay M, Iriarte J, Ederra I, Gonzalo R, de Maagt P (2007). Thin AMC structure for radar cross-section reduction. IEEE Trans. Ant. & Prop..

[24] Pan W (2016). Combining the absorptive and radiative loss in metasurfaces for multi-spectral shaping of the electromagnetic scattering. Sci. Rep..

[25] Zhang Y, Mittra R, Wang B, Huang N (2009). AMCs for ultra-thin and broadband RAM design. Electron. Lett..

[26] Iriarte Galarregui JC (2013). Broadband radar cross-section reduction using AMC technology. IEEE Trans. Ant. & Prop..

[27] Edalati A, Sarabandi K (2014). Wideband, Wide Angle, Polarization independent RCS reduction using nonabsorptive miniaturized-element frequency selective surface. IEEE Trans. Ant. & Prop..

[28] Chen W, Balanis CA, Birtcher CR (2015). Checkerboard EBG surfaces for wideband radar cross section reduction. IEEE Trans. Ant. & Prop..

[29] Chen W, Balanis CA, Birtcher CR (2016). Dual wide-band checkerboard surfaces for radar cross section reduction. IEEE Trans. Ant. & Prop..

[30] Su P, Zhao Y, Jia S, Shi W, Wang H (2016). An Ultra-wideband and polarization-independent metasurface for RCS reduction. Sci. Rep..

[31] Poli R, Kennedy J, Blackwell T (2007). Particle swarm optimization. Swarm intelligence.

[32] Kennedy, J. & Eberhart, R. A discrete binary version of the particle swarm algorithm. In *IEEE Int. Conf. Systems, Man, and Cybernetics* 4104–4108 (1997).

[33] Jin N, Rahmat-Samii Y (2007). Advances in particle swarm optimization for antenna designs: real-number, binary, single-objective and multiobjective implementations. IEEE Trans. Ant. & Prop..

[34] Kennedy, J. & Eberhart, R. Particle swarm optimization. In *IEEE Int. Conf. Neural Networks* 1942–1948 (1995).

[35] Panda S, Padhy NP (2008). Comparison of particle swarm optimization and genetic algorithm for FACTS-based controller design. Applied Soft Computing.

[36] CST Studio Suite, Computer Simulation Technolog ag., http:www.cst.com. Last accessed: July 27, 2017.

